# Integrative Analysis of the Developing Postnatal Mouse Heart Transcriptome

**DOI:** 10.1371/journal.pone.0133288

**Published:** 2015-07-22

**Authors:** Jingyi Gan, Hans-Joachim Sonntag, Mei kuen Tang, Dongqing Cai, Kenneth Ka Ho Lee

**Affiliations:** 1 Stem Cell and Regeneration Thematic Research Programme, School of Biomedical Sciences, Chinese University of Hong Kong, Shatin, N.T., Hong Kong, Hong Kong; 2 Medical Research Council Human Genetics Unit, Medical Research Council Institute of Genetics and Molecular Medicine, University of Edinburgh, Edinburgh, EH4 2XU, United Kingdom; 3 Key Laboratory for Regenerative Medicine, Ministry of Education, Ji Nan University, Guangzhou, 510632, China; Cincinnati Children's Medical Center, UNITED STATES

## Abstract

In mammals, cardiomyocytes rapidly proliferate in the fetus and continue to do so for a few more days after birth. These cardiomyocytes then enter into growth arrest but the detailed molecular mechanisms involved have not been fully elucidated. We have addressed this issue by comparing the transcriptomes of 2-day-old (containing dividing cardiomyocytes) with 13-day-old (containing growth arrested cardiomyocytes) postnatal mouse hearts. We performed comparative microarray analysis on the heart tissues and then conducted Functional annotation, Gene ontology, KEGG pathway and Gene Set enrichment analyses on the differentially expressed genes. The bioinformatics analysis revealed that gene ontology categories associated with the “cell cycle”, “DNA replication”, “chromosome segregation” and “microtubule cytoskeleton” were down-regulated. Inversely, “immune response”, “extracellular matrix”, “cell differentiation” and “cell membrane” were up-regulated. Ingenuity Pathways Analysis (IPA) has revealed that GATA4, MYH7 and IGF1R were the key drivers of the gene interaction networks. In addition, Regulator Effects network analysis suggested that TASP1, TOB1, C1orf61, AIF1, ROCK1, TFF2 and miR503-5p may be acting on the cardiomyocytes in 13-day-old mouse hearts to inhibit cardiomyocyte proliferation and G1/S phase transition. RT-qPCR was used to validate genes which were differentially expressed and genes that play a prominent role in the pathways and interaction networks that we identified. In sum, our integrative analysis has provided more insights into the transcriptional regulation of cardiomyocyte exit from the cell cycle during postnatal heart development. The results also pinpoint potential regulators that could be used to induce growth arrested cardiomyocytes to proliferate in the infarcted heart.

## Introduction

Heart disease is currently one of the leading causes of human morbidity and mortality in developed countries [1]. Coronary heart disease is the most common condition, characterized by atherosclerotic stenosis of the coronary arteries and reduced blood supply to the cardiac muscles. Acute thrombus in one of these coronary arteries could induce myocardial infarction, which is a serious life threatening condition. Myocardial infarction often results in the death of approximately 1 billion cardiomyocytes on the left ventricle within a matter of hours. The damage to cardiac function can be progressive and often leads to congestive heart failure. The inability of adult cardiomyocytes to undergo self-renewal, differentiation and repair is the main reason why myocardial infarction is still untreatable in regenerative medicine.

It has been proposed that cardiomyocytes may be capable of a limited degree of self-renewal in order to repair the daily micro-damages that occur in the adult heart [2–5]**.** This implies that it may be possible to reinitiate cardiomocyte proliferation in the adult heart if we understand the molecular mechanism involved in its growth arrest. In this context, the activation of the intrinsic repair mechanisms of cardiomyocytes has been proposed as a potential means of repairing the heart [6]. Studying the proliferation and growth arrest of cardiomyocytes in the heart during the postnatal period may allow us to understand more about this intrinsic repair mechanisms [7]. We have previously reported that mouse cardiomyocytes proliferate robustly in the 2 day-old neonate heart and that this process was not discernable at day 13 [8, 9]. This finding pinpoints the critical period that will allow us to elucidate the mechanisms and pathways involved in maintaining cardiomyocyte proliferation and their entry into growth arrest. Elucidating the molecular changes involved in inducing cardiomyocyte growth arrest will allow the identification of signaling factors that can trigger cardiomyocyte to reenter into cell proliferation and replace damaged cardiomyocytes in myocardial infarction.

**Fig 1:**
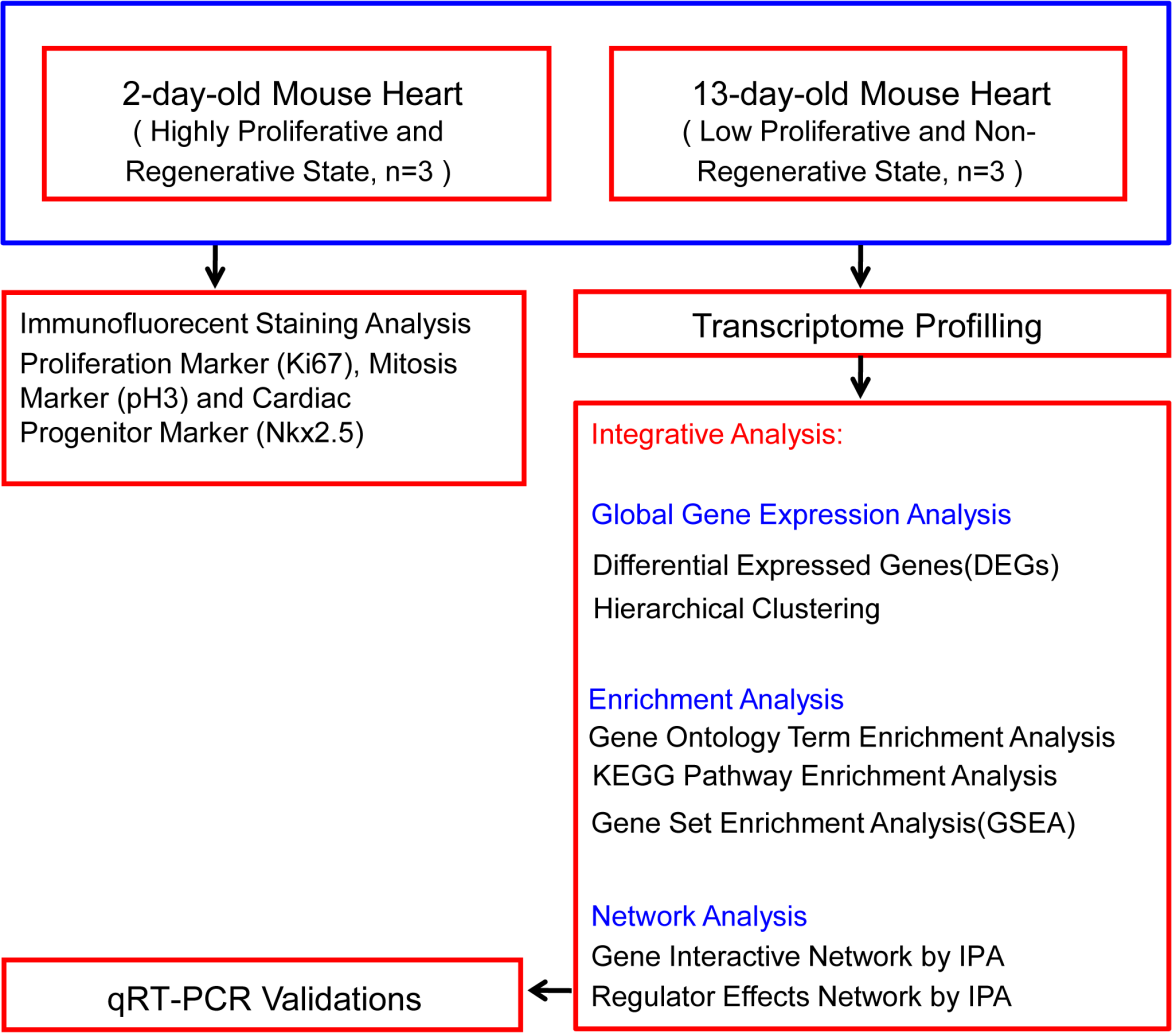
Chart summarizing experiments and analyses conducted in this study.

The present study aim at determining the differences in global gene expression between proliferating cardiomyocytes in the 2-day-old mouse heart and growth arrested cardiomyocytes in 13-day-old heart. Furthermore, functional annotation, gene ontology enrichment, pathway enrichment and interaction network analyses were conducted on the differentially expressed genes (DEGs) identified to gain a comprehensive insight into the signaling pathways involved in regulating postnatal heart development (as shown in Fig 1).

## Materials and Methods

### Animals and tissue preparation

Two- and 13-day-old mouse neonates were obtained from the Laboratory Animal Services Centre, Chinese University of Hong Kong. The neonates were maintained in individually ventilated cages under controlled room temperature (25°C), at constant humidity of 55% and a 12 h dark/light cycle. The neonates were sacrificed by injections of 200 mg/kg ketamine and 10 mg/kg xylazine, followed by cervical dislocation. The hearts from these 2- and 13-day-old neonates were harvested for total RNA isolation immediately after dissection and several washes in DEPC-PBS. All experiments involving the use of animals were approved by the Department of Health (Ref No.:13–2 in DH/HA&P/8/2/1 Pt.28) and Chinese University of Hong Kong Animal Care and User Committee.

### Immunofiuorescent Staining and Microscopy

Two- and 13-day-old mouse hearts were freshly harvested, fixed in 4% paraformaldehyde and dehydrated in 30% sucrose solution overnight [10]. The hearts were then embedded in OCT and sectioned at 5μm on a Cryotome (Thermo Scientific, USA). The sections were mounted onto glass slides and then washed twice in PBS for 10 min. For immunofiuorescent staining, the sections were first permeabilized in 0.3% Triton X-100 and 0.1% SDS in PBS solution for 30 min, followed by blocking in 2% BSA plus 5% donkey serum for 1 h. The sections were then stained overnight at 4°C with the following primary antibodies diluted in blocking solution (1:200): Mouse anti-Cardiac Troponin T antibody (cTnT, Abcam), Rabbit Ki-67 antibody (Abcam), Rabbit pH3 antibody (Santa Cruz) and Rabbit Nkx2.5 antibody (Abcam). The tissues were washed three times with PBST (PBS buffer with 0.05% Tween 20) for 15 min to remove all unbound primary antibodies. Then the appropriate secondary antibody (1:300 dilution) conjugated to Alexa Fluor-488, -555 or -647 (Life Technologies) was added to the tissues for 1 hr at room temperature, in the dark with gentle shaking. The unbound secondary antibody was removed by washing with PBST, three times for 10 min and PBS for 5 min. The cell nuclei were counter-stained with 4, 6-diamidino-2-phenylindole (DAPI, Molecular Probes) in 70% (v/v) glycerol and mounted with glass cover slips. The immunofiuorescent staining were captured under a confocal microscope (FV1000; Olympus, Japan) with fixed exposure settings for all of the samples [11].

### Comparative microarray analysis

Total RNA was isolated from 2- and 13-day-old hearts using an RNeasy Fibrous Tissue Mini Kit (Qiagen, USA), according to the manufacturer’s instructions. The RNA concentration and quality were assessed using a Nanodrop ND-1000 Spectrophotometer with absorbance set at 260 nm and ratio of 260/280 (Nanodrop Technologies, Wilmington, DE). The RNA integrity was further checked on a 1% agarose gel. 10 μg of the total RNA was reverse transcribed into cDNA using a SuperScript Double-Stranded cDNA Synthesis Kit (Invitrogen, USA). The resulting cDNA samples were labelled with Cy3 random nanomers using the NimbleGen One-Color DNA labelling kit (Roche NimbleGen, Madison, WI, USA) according to manufacturer’s instruction. The transcriptome profiles of the 2- and 13-day-old hearts were analyzed by direct comparison of the transcriptional level between these two groups on the same 12×135K microarray (Roche NimbleGen). Hybridization was performed on the microarray at 42°C for 16 h and the microarray cassette was washed using a NimbleGen wash buffer kit. Then, the microarray was scanned using a NimbleGen MS 200 Microarray Scanner, which produced 532 nm TIFF images. The scanned images were imported into the DEVA software (Roche) to quantify the signal intensities of corresponding probes in the microarray [11, 12]. The microarray data has been deposited in Gene Expression Omnibus (http://www.ncbi.nlm.nih.gov/geo/) number GSE67575.

### Bioinformatics analysis of differentially expressed genes

The raw data generated from the NimbleGen microarrays were transformed using the robust multi-array average method. This widely used algorithm results in a background-adjusted and log-transformed expression value that has been normalized across samples using quantile normalization and summarized from probe to transcript level using the median polish algorithm [13]. Genes that are differentially expressed in 2- and 13-day-old mouse hearts were identified via one-way ANOVA using the Partek Genomics Suite (Partek, Inc., St. Louis, MO). To adjust for multiple comparisons, we used the Benjamini-Hochberg step-up procedure to calculate the false discovery rate (FDR). Our thresholds for identifying positive differential gene expression were taken as FDR < 0.05 and fold change ≥ 1.5. Hierarchical clustering analysis was performed on the DEGs using Partek default settings.

### Gene Ontology & KEGG pathway enrichment analysis

A list of differentially expressed genes in 2- versus 13-day-old hearts was imported into the Database for Annotation, Visualization, and Integrated Discovery (DAVID) Bioinformatics Resource (http://david.abcc.ncifcrf.gov/). The aim was to detect significantly over-represented biological processes affected by changes in the transcriptomes. The David Functional Annotation Tool [14] provides extended annotation coverage drawn from over 40 databases, including GO terms, disease associations, pathways, gene functional summaries, gene tissue expression, and literatures. Functional enrichment was assessed using the following four annotation databases including biological process (BP), molecular function (MF), cellular component (CC) and KEGG pathway by selecting ‘GOTERM_BP_ALL’, ‘GOTERM_MF_ALL’, ‘GOTERM_CC_ALL’ and ‘KEGG_PATHWAY’.

### Gene Set Enrichment Analysis (GSEA)

To validate the findings from DAVID and confirm that they are robust to our choice of significance threshold, we have performed GSEA for the enrichment of KEGG pathways. This method considers the full list of genes, ranked by their correlation with phenotype, and calculates an enrichment score to determine whether members of a particular pathway are randomly distributed or non-randomly enriched at either ends of the ranked list. We chose the t-test as metric for ranking genes and 1,000 gene set permutations were used to generate a null distribution for the enrichment score, which was used to yield a normalized enrichment score (NES) for each pathway. Gene sets were only considered significantly enriched if p < 0.05 and FDR < 0.25 [15].

### Interaction network analysis by IPA

We examined the molecular interactions that were not captured by previously characterized and well curated pathways. Hence, the differentially expressed genes were further analyzed using the Ingenuity Pathways Analysis (IPA) software (Ingenuity Systems, Redwood City, CA; http://www.ingenuity.com). This all-in-one web-based software which makes use of the Ingenuity Pathways Knowledge Base (IPKB) to generate interaction networks of focus genes based on manually curated information reported in the literatures. The underlying algorithm maximizes connectivity, leading to networks that are likely to represent significant biological function. Briefly, we uploaded a file containing gene identifiers (ID), their corresponding fold change and p-values. We also specified mouse as the species and heart as the tissue. Focus genes were selected by a 1.5 fold cutoff change. Enrichment of the focus genes in the networks (which always consist of 35 genes) were assessed via Fisher’s exact test and used to rank the networks. Furthermore, the software identifies top functions and diseases associated with each network via enrichment scores, highlighting the biological significance of the results.

### Regulator effects analysis using Ingenuity Knowledge Base

To gain insight into the causes and effects of DEGs in the dataset, we performed regulator effects analysis using the Ingenuity Knowledge Base**.** The analysis produces causal hypotheses that predicted and explain how activation or inhibition of upstream regulators might cause an increase or decrease in phenotypic and functional outcomes downstream. A top scoring interactive network was established from the Regulator Effects analysis of DEGs using the Ingenuity Knowledge Base. In the network analysis, only genes, RNAs, proteins, microRNAs and growth factors were selected as upstream regulators. We also required that the regulators and downstream effects were fed into the algorithm with an absolute z-score of >2 and a p-value <0.05.

### Quantitative Real-time Polymerase Chain Reaction (RT-qPCR) assay

Total RNA was extracted from heart tissues using a NucleoSpin Total RNA Isolation Kit (Clontech Laboratories, USA) according to the manufacturer’s instructions. After DNase I (Takara, Japan) digestion for 15 min, the RNA concentration was determined using a NanoDrop 2000 Spectrophotometer (Thermo Fisher Scientific, USA). 1 μg of total RNA was reverse-transcribed into cDNA with oligo (dT_18_) primer using a RevertAid First Strand cDNA Synthesis Kit (Thermo Scientific, USA) according to manufacturer’s instructions. The reaction mixture (20 μL) was incubated for 60 min at 42°C, for 5 min at 70°C and then stored at -20°C. SYBR Green Master Mix Kit was used in the RT-qPCR to quantify the expression of key genes extrapolated from the microarray analyses. PCRs were performed in triplicate using an ABI 7900HT Real-Time PCR Detection Systems (Applied Biosystems, USA) and 2×SYBR Premix Ex Taq (Takara, Japan) at 95°C for 30s and 40 cycles at 95°C for 5s and 60°C for 30s. After RT-qPCR amplification, a dissociation (melting curve) analysis was performed to identify the characteristic peaks associated with primer-dimers in order to separate them from the single prominent peak which represents the successful PCR amplification of mRNA. Relative expression level of a given mRNA transcript was determined by delta-delta (△△) Ct calculation with GAPDH as the reference housekeeping gene. Expression levels of mRNA were presented as fold changes $\left(2^{{-}^{△△}\text{Ct}}\right)$ and represented as mean ± standard deviation, obtained from three different samples. A t-test was performed to compare the expression in 2- and 13-day-old hearts, with p < 0.05 as being significance different. The primer sequences for validating 5 of the top 10 down-regulated genes (BMP10, Hand1, Hif3a, Myo3b and IGF2bp1), 5 of the top 10 up-regulated genes (Hmcn2, Mapt, Art1, Itgb6 and Rxrg), 6 genes from the most significantly enriched pathway (c-Myc, CycA, CycD, CycE, CDK1 and GADD45) and 6 genes from the top IPA network (GATA4, MYH7, IGF1R, IGF1, MEF2D and TNNI) are all listed in **S1 Table.**

### Statistical Analysis

All data presented were obtained in triplicates. The results were calculated and analyzed using GraphPad Prism computer software and presented as mean ± standard deviation (SD). Comparison of differences between data sets was determined by Student’s t-test and analysis of variance (ANOVA).

## Results

### Cardiomyocyte proliferation and differentiation in postnatal mouse hearts

**Fig 2:**
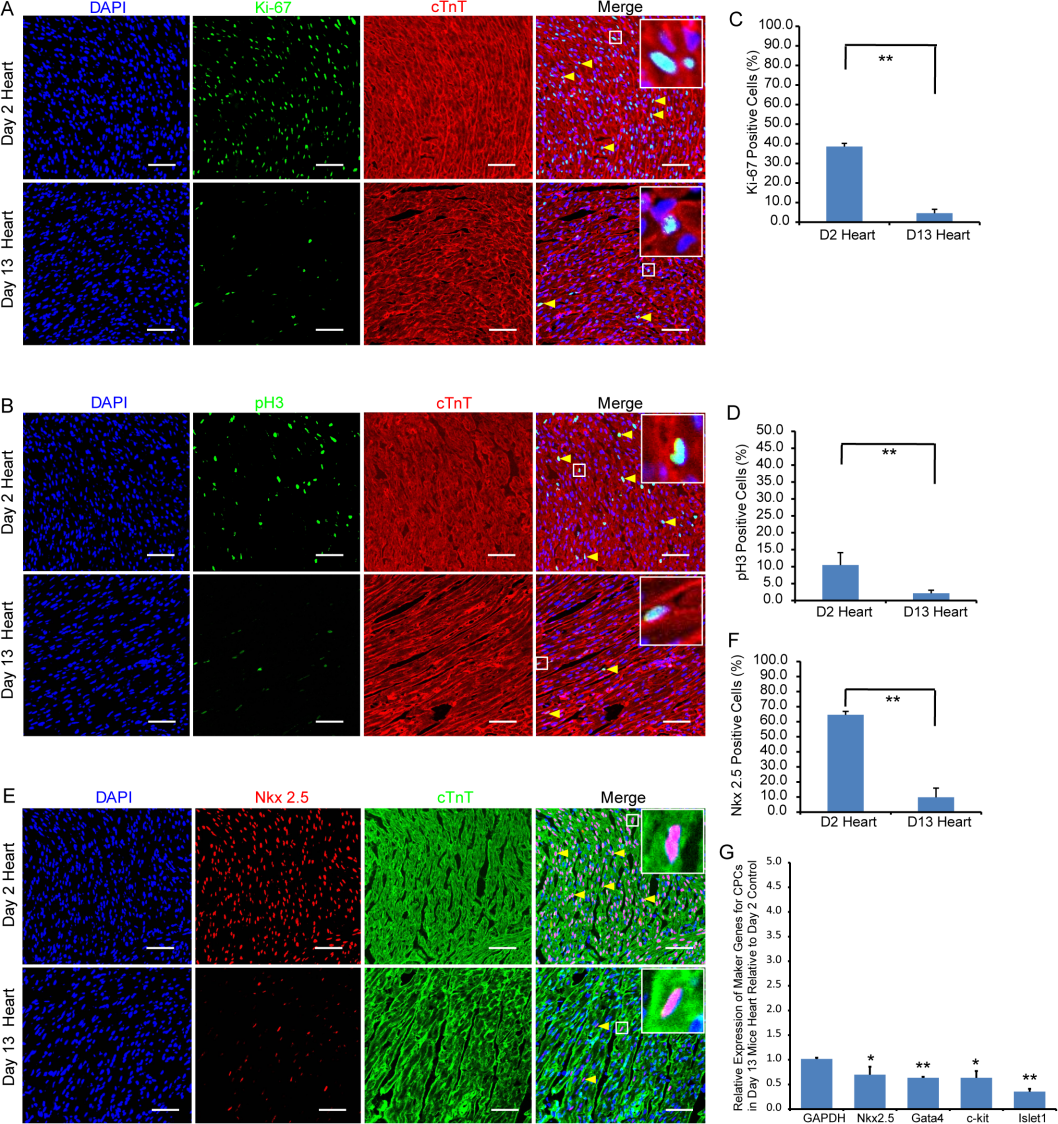
Extent of cardiomyocyte proliferation and differentiation in 2- and 13-day-old mouse hearts. Immunofluorescent staining showing the presence of proliferating Ki-67^+^ (A) and mitotic pH3^+^ (B) cTnT^+^ cardiomyocytes (yellow arrow heads) at day 2 and day 13. (E) showing the presence of Nkx2.5^+^ cardiac progenitor cells at day 2 and day 13. Bar charts showing the percentage of cardiomyocytes that are Ki-67^+^ (C), pH3^+^ (D) or Nkx2.5^+^ (F) in 2- and 13-day-old hearts. The data is presented as means ± SD; Scale bar: 50μm, Significantly different: **P<0.01. (G) RT-qPCR assay confirms the immunofluorescent staining that Ki-67^+^ and pH3 expressions are down-regulated in 13-day-old heart. Relative expression values are presented as means ± SD. Significantly different: *P<0.05, **P<0.01.

To establish the extent of cardiomyocyte proliferation in 2- and 13-day-old hearts, we stained these sections of organs with antibodies for Ki-67 (a cell proliferation marker) and phosphorylated Histone H3 (pH3, a mitosis marker). We quantitatively determined that 38.5 ± 1.6% of cardiomyocytes was Ki-67^+^ in 2-day-old hearts, while only 4.6 ± 1.9% were Ki-67^+^ in 13-day-old hearts (**Fig 2A and 2C**). Similarly, we determined that there was significantly fewer pH3^+^ cardiomyocytes in 13-day than 2- day-old hearts (2.1 ± 0.9% vs 10.4 ± 3.7%, respectively **Fig 2B and 2D**). The results show that there was a significant reduction in cardiomyocyte proliferation and mitosis in the 13-day-old heart. To determine the extent of cardiomyocyte differentiation, we immunofluorescently stained the hearts with Nkx2.5 antibodies. Nkx2.5 is a transcription factor that is normally expressed in cardiac progenitor cells. We determined that 64.5 ± 2.3% of cardiomyocytes was Nkx2.5^+^ in 2-day-old hearts verses 9.8 ± 6.1% in 13 day (**Fig 2E and 2F)**. RT-qPCR analysis also revealed that expression of other cardiac progenitor cell markers, including Gata4, c-kit and islet1, were correspondingly down-regulated at day 13 (**Fig 2G**). These results imply that cardiomyocytes in the heart starts to undergo growth arrest and differentiation during the postnatal period.

### Microarray analysis of 2- and 13-day-old hearts

**Fig 3:**
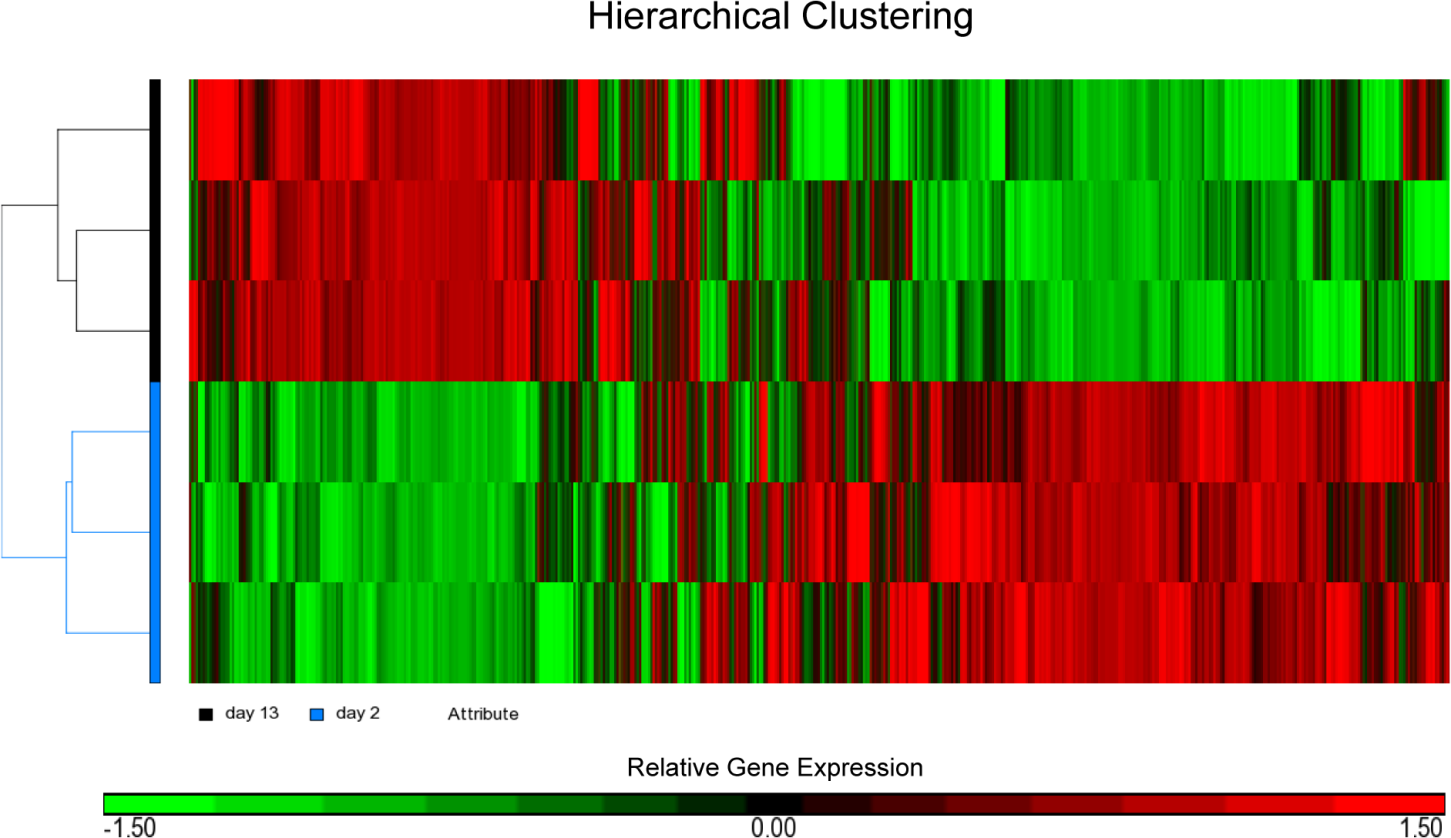
Hierarchical clustering of the microarray results showing the presence of two distinct heart samples. Relative level of gene expression is color-coded: red implies genes up-regulated in 13-day-old heart while green indicate genes that were down-regulated.

We used NimbleGen microarray chips to generate the transcriptome profiles of 2- and 13-day-old mouse hearts. Using a significance threshold of FDR < 0.05 and fold change ≥1.5, we determined there were 3,273 transcripts that were differentially expressed between the 2- and 13-day-old hearts (**S2 Table**). Out of these 3,273 differentially expressed transcripts, 1,768 of them were determined to be up-regulated while 1,505 were down-regulated. Hierarchical clustering analysis, based on all genes after normalization, revealed there was a clear separation between samples from 2- and 13-day-old hearts. This indicates that such changes are robust different between the two groups and not strongly affected by biological variation within the groups (**Fig 3**).

### Functional annotation and gene ontology enrichment analysis

**Fig 4:**
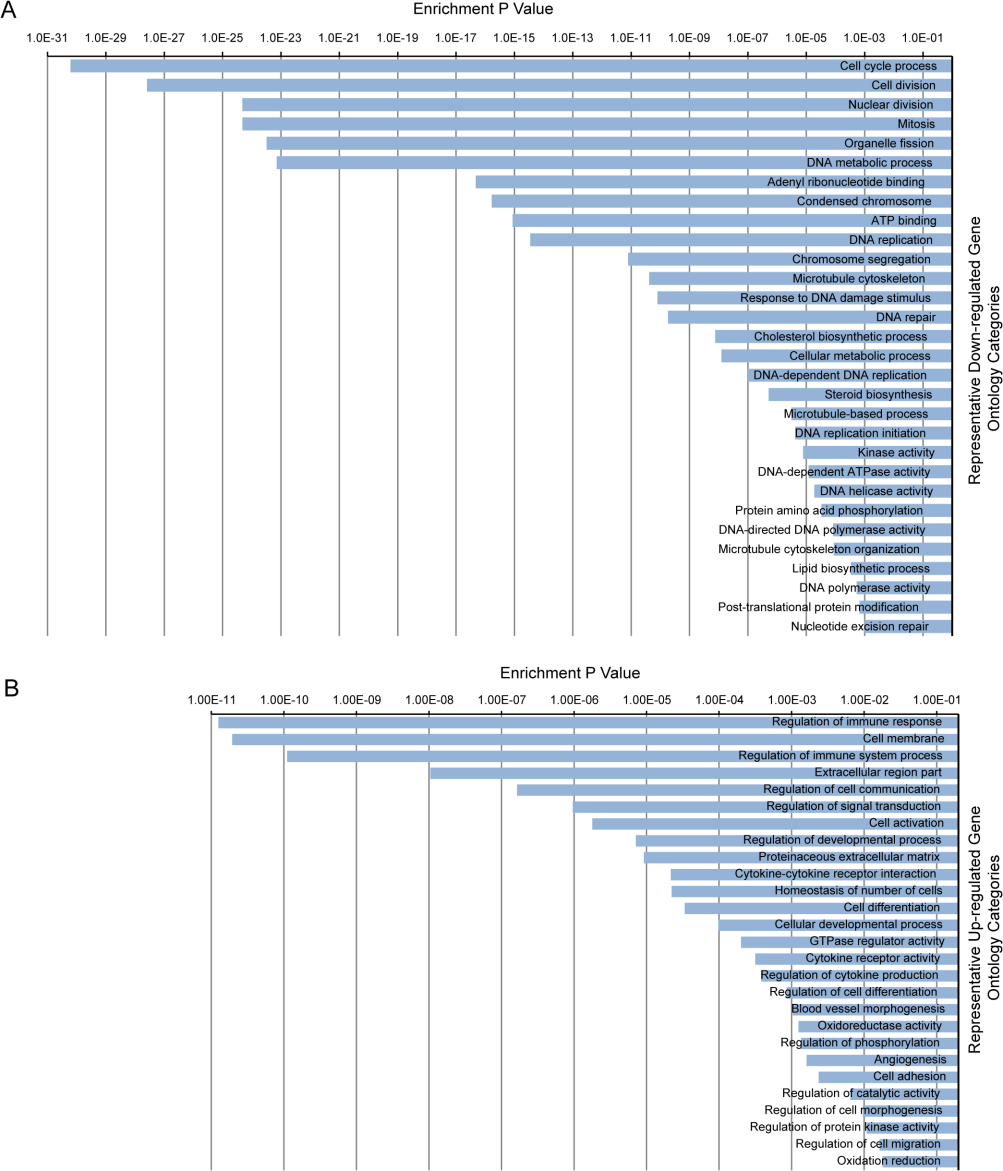
Gene Ontology enrichment analysis showing representative ontology categories. (A) Representative down-regulated ontology categories. (B) Representative up-regulated ontology categories. The enrichment p value is displayed on the x-axis.

We performed functional batch annotation and ontology enrichment analysis on the DEGs that we identified to understand the global transcriptional changes that occur between 2- and 13-day-old hearts. We identified important representative gene ontology categories based on annotation of 4 databases that included: biological process, molecular function, cellular component and KEGG pathway. The gene ontology categories identified were related to the cell cycle, chromosome segregation, microtubule cytoskeleton, DNA metabolic process, cell membrane, extracellular matrix, cell differentiation, cytokines production and immune response (**Fig 4A and 4B**). The total annotation clusters that were determined to be down-regulated or up-regulated are shown in **S3A and S3B Tables**, respectively. These results suggest that cell cycle progression and DNA replication were inhibited in 13-day-old hearts while the immune response and differentiation process were correspondingly enhanced.

### KEGG pathway mapping analysis

**Fig 5:**
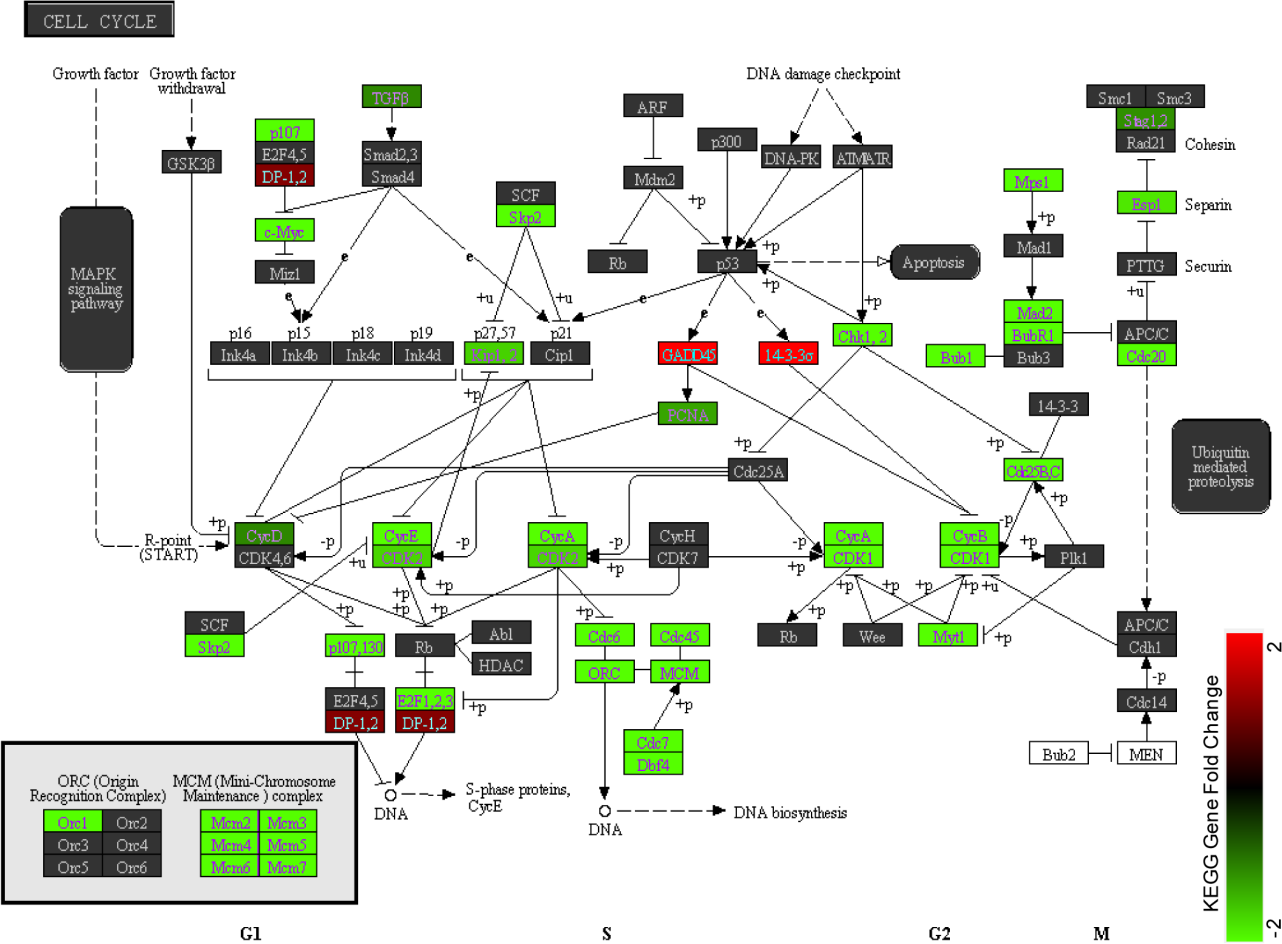
KEGG pathway map of the cell cycle. Cell cycle associated genes are significantly enriched in the list of differentially expressed genes (p = 8.6 x 10^−9^). Fold change is only shown for genes within the list and illustrates near universal down-regulation in the pathway for cardiomyocytes in the 13-day-old heart, as compared with the 2-day-old heart. Arrows indicate positive regulation, while other lines indicate negative regulation.

We conducted KEGG enrichment analysis and identified 41 pathways that were significantly enriched among the DEGs (P<0.05, **S4 Table**). The top 2 enriched KEGG pathways identified were “cell cycle” (p = 9.31×10^−9^) and “DNA replication” (p = 3.0×10^−8^). Closer examination of the pathway maps confirmed the inhibition of these two biological processes, as most of the genes in the pathways were down-regulated (as highlighted in green in **Fig 5**) in 13- versus 2-day-old hearts. Specifically, the cell cycle arrest may be attributed to five mechanisms as evident in the pathway map. The five sets of genes inhibited from being expressed includes: 1) Cyclin genes (CycA, CycB, CycD and CycE)*;* 2) cyclin-dependent kinases (CDK1 and CDK2); 3) cell cycle-related genes (CDC6, CDC7, CDC20, CDC45, MCM2-7 and c-Myc); 4) CDK interacting protein/Kinase inhibitory protein, especially p27^kip1^ and p57^kip2^ (which activate the cyclin-dependent kinase inhibitors and arrest cells in G1 phase) and 5) BUB1 and BUBR1 (mitotic checkpoint serine /threonine-protein kinases).

We have determined that genes involved in various metabolic pathways were also significantly enriched (**S4 Table).** These pathways involved: steroid and fatty acid biosynthesis, glutathione metabolism, synthesis and degradation of ketone bodies, terpenoid backbone and primary bile acid biosynthesis. In addition, we have identified pathways relating to the immune system (such as antigen processing and presentation, allograft rejection, and graft-versus-host disease) were all well represented. Cardiac disease related pathways were also flagged up and these included hypertrophic cardiomyopathy, dilated cardiomyopathy and arrhythmogenic right ventricular cardiomyopathy. These findings point to a complex interacting network of growth- and metabolism-related changes that occur during the crucial period (postnatal day 2 to 13) of heart development. Significantly, components in these networks may represent potential targets for inducing adult cardiomyocytes to re-enter the cell cycle when cardiac repair is required.

### Gene set enrichment analysis confirms inhibition of the cell cycle and DNA replication

**Fig 6:**
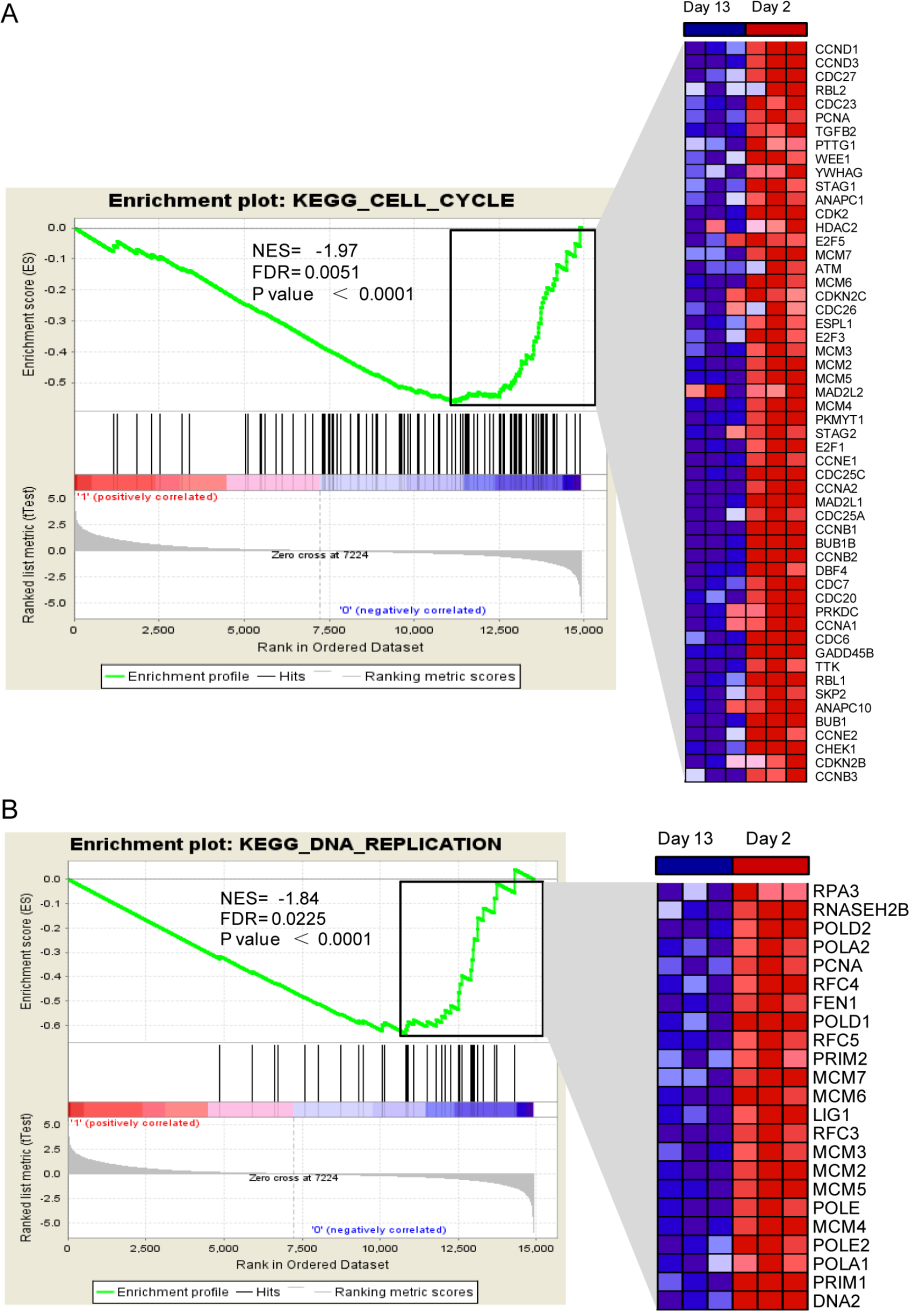
Gene Set Enrichment Analysis (GSEA) of 13-day-old versus 2-day-old mouse hearts. GSEA analysis reveals that there is significant inhibition of the (A) cell cycle and (B) DNA replication gene sets. On the x-axis, the mouse genes are ranked from the most up-regulated (left end) to the most down-regulated (right end) between 2- and 13-day-old hearts. The “barcode” indicates the position of the genes from the biological pathway. The y-axis shows a running enrichment score (ES), which goes up when genes are encountered in the pathway and goes down otherwise—leading to an assessment of the distribution within the set of all genes. The normalized enrichment score (NES) is inferred from permutations of the gene set and the false discovery rate (FDR). P values are indicated and the plot on the right shows the relative level of gene expression (red = high, blue = low) in the leading edge subset, corresponding to the most significant genes.

Using GSEA, we took a closer examination of the distribution of KEGG pathway gene sets, in a ranked list of up- and down-regulated genes. Altogether, there were 73 down-regulated gene sets that were scored as enriched and approximately 14 of these were statistically significant (**S5 Table**). In agreement with the results from our simple enrichment analysis, the data sets with the highest statistical significance were the cell cycle and DNA replication sets, which are both refiecting the growth arrested phenotype of adult cardiomyocytes. For these two data sets, the false discovery rate q-values were 0.0051 and 0.0225, respectively and similar trends in gene activation were observed between 2- and 13-day-old hearts (**Fig 6)**.

### Top gene interaction network elucidated from Ingenuity Pathways Analysis

**Fig 7:**
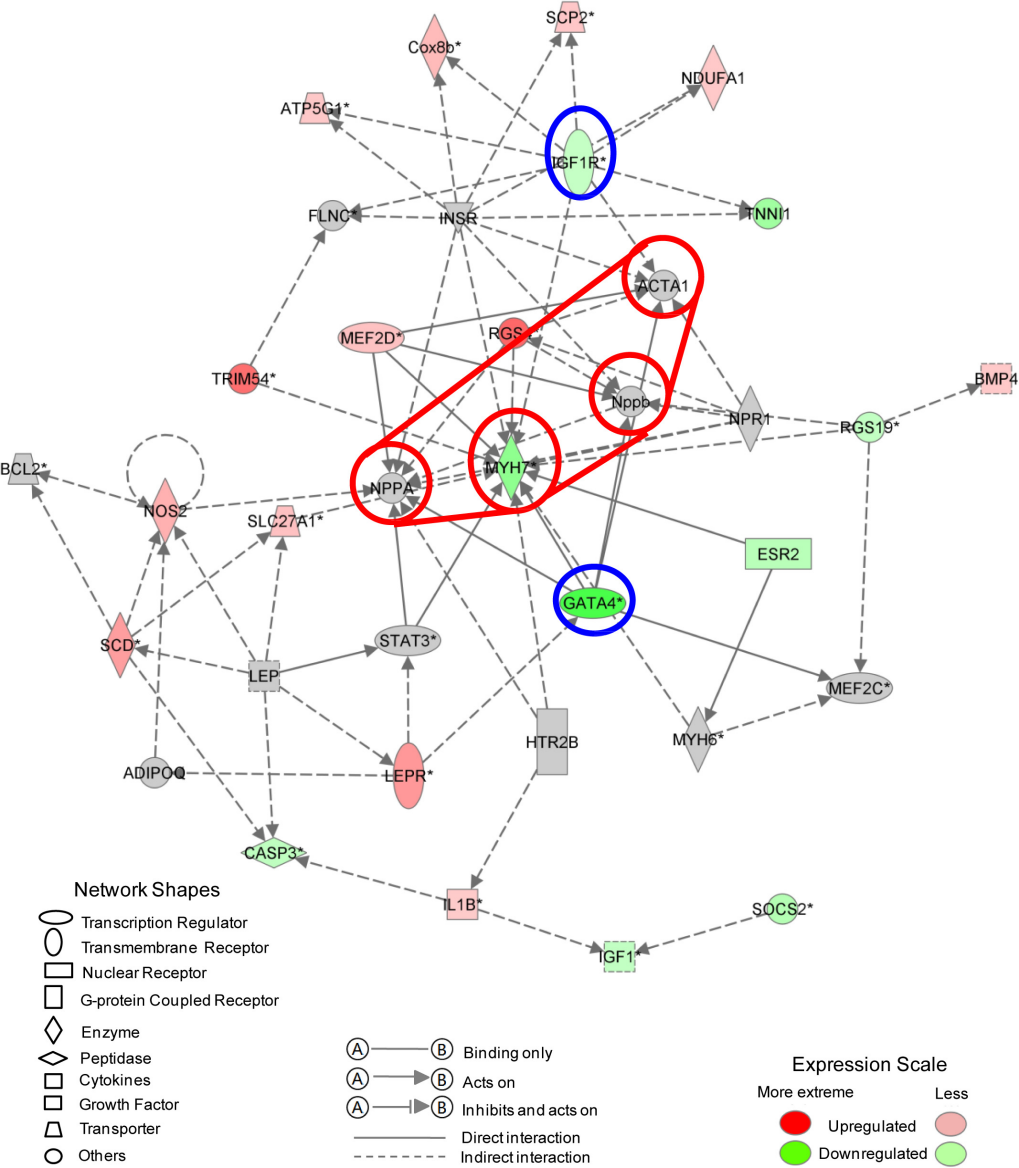
Top gene interaction network based on information from the Ingenuity Pathways Knowledge Base. Genes that are up- or down-regulated are labelled in red and green, respectively. Genes that are colored gray are not differentially expressed between Day 2 and Day 13.

We algorithmically generated a gene interaction network based on the connectivity of focused genes (together with other related genes or molecules strongly inferred as interacting partners by IPA). In the diagram, genes and molecules are depicted as nodes while interactions (supported by at least one reference from the literature or textbook stored in IPKB) are illustrated by the edges connecting them. The nodes are displayed as various shapes that represent their functional classification while the different lines describe the types of relationship. Notably IPA uses a solid line to indicate direct interactions while a dashed line denotes an indirect interaction between two nodes. **Fig 7**shows the IPA network with the highest enrichment of focused genes. The top five associated functions identified were related to cardiac hypertrophy, cell cycle, cardiovascular disease, cardiovascular system development and cardiac fibrosis, as illustrated in **S6 Table**.

Since IPA networks indicate directionality, we could focus on the nodes with the highest indegree (number of incoming edges) and outdegree (number of outgoing edges). These are highlighted in red and blue, respectively (**Fig 7**). The first group consists of MYH7, NPPA, NPPB and ACTA1 implying that the regulation of these genes, together with downstream effects, plays an important role in shifting the cardiomyocytes towards a lower rate of cell proliferation. More importantly, GATA4 and IGF1R have the highest outdegree, with GATA4 directly regulating all of the four central nodes mentioned above. Both of these crucial genes have previously been reported to play an important role in cardiomyocyte proliferation and regeneration [16–24]. Based on our analysis, we envisage that activation of pathways such as the IGF1 signaling pathway or up-regulation of the key regulator GATA4 may induce cell cycle re-entry and facilitate cardiac repair.

### Regulators effect network generated from Ingenuity Pathways Analysis

**Fig 8:**
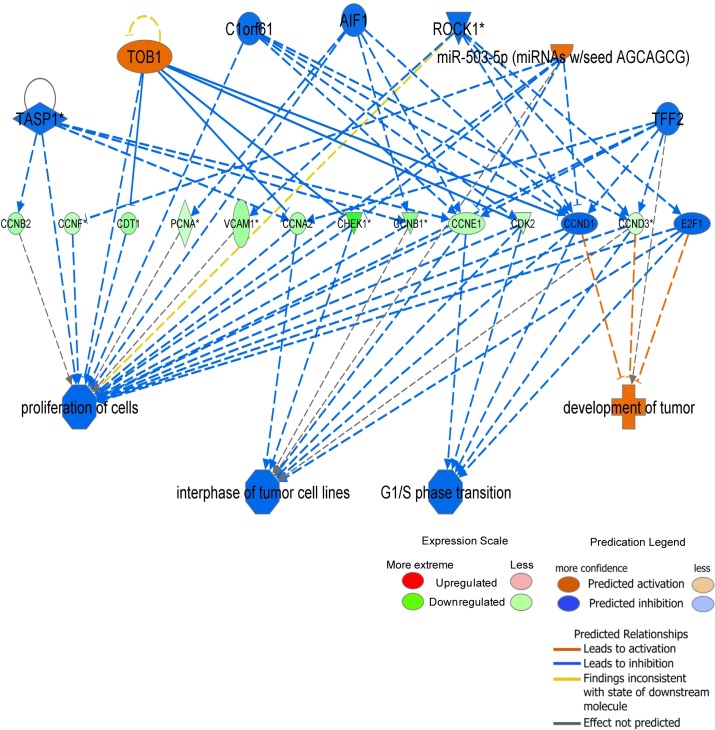
Regulator effects network generated from Ingenuity Pathways Analysis. Upstream regulators are displayed in the top tier, while diseases, functions and phenotypes are displayed in the bottom tier. The dataset genes that connect the upper regulators and the lower diseases and functions are displayed in the middle tier. The causal consistency relationships are represented with orange and blue lines, while the inconsistent relationships are represented as yellow line. Note that some of the dataset genes displayed in the middle tier may have been predicted to be activated or inhibited by upstream regulators, and under such situation is colored orange or blue, respectively (for example CCND1 and E2F1 in the network).

Regulator Effect network, which integrates the upstream regulator results with the downstream effects results, was used to generate a cause-and-effect hypothesis **(Fig 8**). The analysis could explain how upstream regulators may cause particular phenotypic and functional outcomes downstream. This network analysis predicated several novel regulators, which includes TASP1, TOB1, C1orf61, AIF1, ROCK1, TFF2 and miR503-5p that may be acting on the 13-day-old heart. These interactions could inhibit cardiomyocyte proliferation and G1/S phase transition by targeting the relevant cell cycle genes in our dataset.

### Validation of gene expression by RT-qPCR Assay

**Fig 9:**
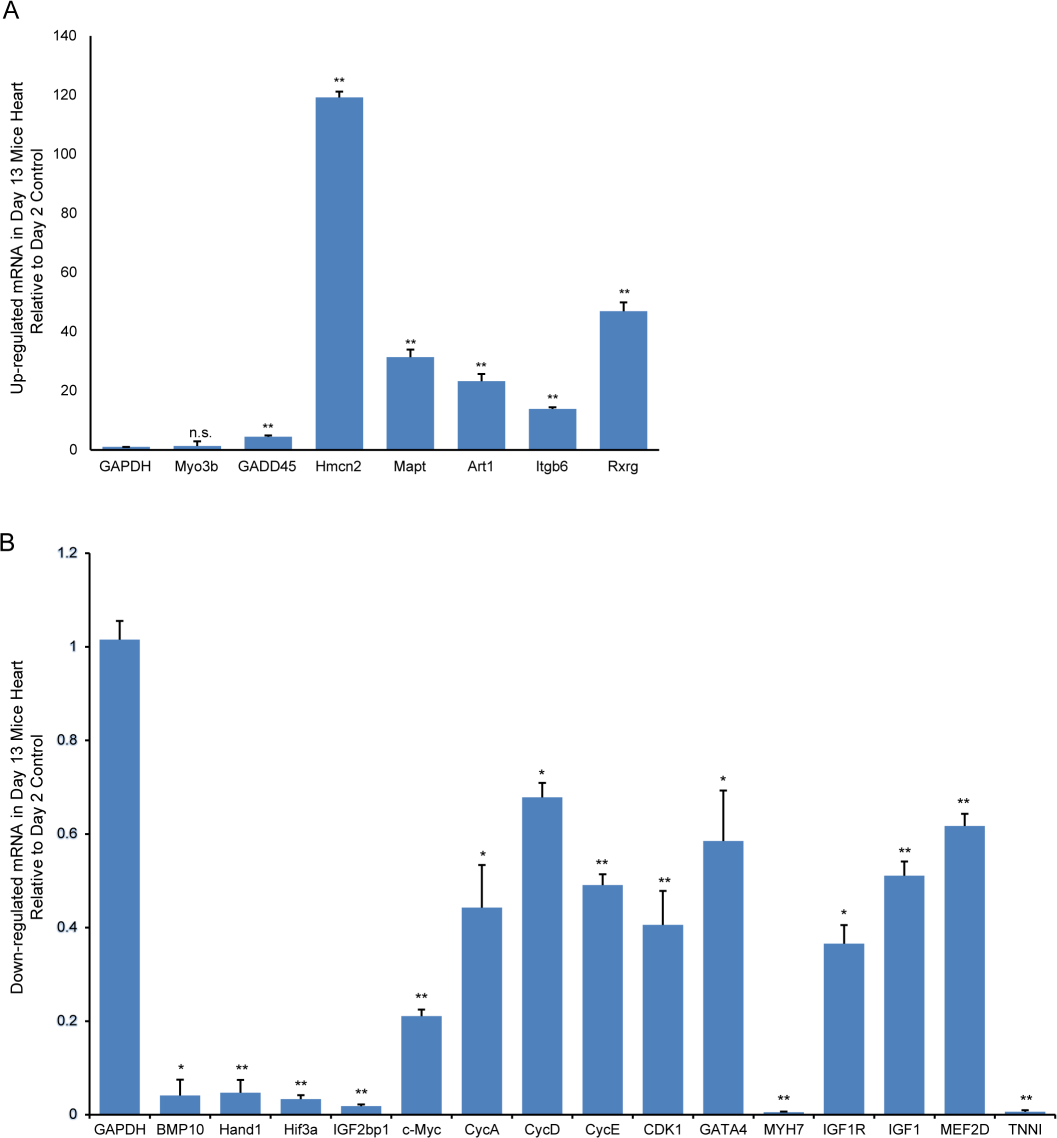
RT-qPCR validation of representative differentially expressed genes. Up- (A) and down-regulated (B) in 13-day-old versus 2-day-old mouse hearts. Relative expression values are presented as means ± SD. Significantly different: *P<0.05, **P<0.01, No significance: n.s.

We selected representative differentially expressed genes from our microarray analysis for validation by RT-qPCR. The genes included: (1) CycA, CycD, CycE, CDK1, c-Myc and GADD45 which are key components of the cell cycle pathway; (2) GATA4, MYH7, IGF1R, IGF1, MEF2D and TNNI in the top IPA network (on account of their degree of relevance in the network or reported functions in cardiomyocyte proliferation and regeneration); (3) BMP10, Hand1, Hif3a, Myo3b and IGF2bp1 which are among the top 20 down-regulated genes (based on their established roles in cardiogenesis) and (4) Hmcn2, Mapt, Art1, Itgb6 and Rxrg in the top 20 up-regulated gene list (selected because these are novel genes in postnatal heart development). For cell cycle pathway-associated genes, RT-qPCR assays revealed that CycA, CycD, CycE, CDK1 and c-Myc expressions were significantly down-regulated in 13-day-old hearts compared with expression in 2-day-old hearts. As expected from the microarray data, GADD45 was significantly up-regulated **(Fig 9)**. For selected genes from the top IPA network, the RT-qPCR showed GATA4, MYH7, IGF1R, IGF1, and TNNI expressions were significant down-regulated in 13- versus 2-day-old hearts. Furthermore, BMP10, Hand1, Hif3a and IGF2bp1 expressions were also significantly down-regulated, while Hmcn2, Mapt, Art1, Itgb6, and Rxrg expressions were significantly up-regulated in 13- versus 2-day-old hearts **(Fig 9)**.

## Discussion

The mammalian heart possesses remarkable regenerative capacity for several days after birth. It then rapidly loses this regenerative ability when the majority of cardiomyocytes “permanently” exit from the cell cycle [25–29]. This exit is accompanied by the cardiomyocytes increasing their cell size via hypertrophy so that the overall mass and size of the heart could increase [30]. Presently, we have demonstrated that the cardiomyocytes undergo dramatic changes, in terms of their ability to proliferate and mature, between day 2 and day 13 of postnatal heart development. During this critical period, our immunofluorescent staining results revealed that cardiomyocyte proliferation steadily declines while cardiomyocyte maturation increases. To elucidate the molecular events and understand the changes that occur during this key period, we performed microarray to generate the transcriptomes for 2- and 13-day-old mouse hearts. We performed Functional annotation, Gene ontology, KEGG pathway and Gene Set enrichment analyses on the two sets of microarray data generated. The analyses revealed that “cell cycle” and “DNA replication” were featured the most prominently annotations amongst the down-regulated genes. This finding collaborates with our immunohistological observations that majority of the cardiomyocytes exit the cell cycle in the 13-day-old heart. These results are highly consistent with findings that we and other have previously reported [5, 31, 32].

Another interesting finding is the prominence of the immune-related GO categories for enrichment of the up-regulated genes. This may be explained by the fact that a newborn mouse has to transit from a sterile intrauterine to an external environment, which is a major pathological event. This event may trigger a stronger role for the immune system so that cardiomyocytes can face the challenges presented by the external environment [33]. Alternatively, the immune system may crosstalk with pathways that regulate cell proliferation to control cardiomyocyte growth and differentiation. We have identified several important gene targets that can be seen as key players in regulating cardiac proliferation and differentiation. In the cell cycle pathway, cyclin A was one of the genes identified as most significantly down-regulated. Normally, cyclin A forms a complex with its cyclin-dependent kinase partners to mediate in both the G1—S and G2—M transitions of the cell cycle. This gene is normally silenced in mammalian cardiomyocytes shortly after birth. Therefore, re-initiating the expression of this gene may be a crucial step in re-establishing the proliferative ability of cardiomyocytes [34, 35].

One important finding from the IPA analysis is the identification of GATA4 and MEF2C in the top network. It has been reported that these genes can elicit cardiomyocyte regeneration *in vivo* following myocardial infarction, induced by ligation of the left anterior descending artery [36, 37]. In related experiments, cardiac fibroblasts at the infarct site have been transfected with retroviruses carrying Gata4, Mef2c and Tbx5. These transfected fibroblasts directly transdifferentiated into functional cardiac-like myocytes, leading to a reduction in the infarct size, improved cardiac function and a reduction of adverse effect from ventricular remodeling for up to 3 months. The newly-formed cardiomyocytes were established around the borders of the infarct site. Furthermore, both studies also revealed that this direct reprogramming of the cardiac fibroblasts was significantly more efficient *in vivo* than under *in vitro* tissue culture conditions. However, these claims have been recently contradicted [38]. It was reported that Gata4, Mef2c and Tbx5 overexpression in cardiac fibroblasts was inefficient at inducing the molecular signature and electrophysiological activities associated with mature cardiomyocytes.

O'Meara et al [39] have profiled the global gene expression patterns of mouse cardiomyocytes during differentiation. They examined the cardiomyocytes on postnatal day 0, 4, 7 and weeks 8–10 to identify genes and gene networks that change dynamically during the differentiation processes. They suggested that cardiac regeneration involves a transcriptional reversion of the cardiomyocyte differentiation process. Furthermore, Giudice et al. [40]showed that extensive transcriptional changes and alternative splicing occurred in the heart between postnatal day 1 and 28. They demonstrated that alternative splicing played a role in vesicular trafficking and intracellular membrane organization. Their findings are consistent with our current data from the gene ontology enrichment analysis. Our reliance on a bioinformatics approach in the current study cannot directly verify the functional relevance of the key genes and regulators that we have identified. For functional follow-up study in the future, we will deliver and over-express adeno-associated virus carrying Gata4, Myh7, TNNI, Hand1, Hif3a and Cyclin A genes into cardiac fibroblasts that occupy the heart infract site. This could be combined with local administration of IGF1 and BMP10 to enhance cardiac regeneration, based on the findings from our results. Theoretically, over-expression of these factors might be able to promote the proliferation of adult cardiomyocytes and also directly reprogram cardiac fibroblasts into new cardiomyocytes.

## Supporting Information

S1 Table Primers of Genes Selected for Validation with RT-qPCR. (XLSX)

S2 Table Differentially Expressed Gene List for 13- versus 2-day-old Hearts. (XLSX)

S3 Tables A, B: Down- and up-regulated Gene Ontology Categories. (XLSX)

S4 Table KEGG Pathways. (XLSX)

S5 Table Gene Set Enrichment Analysis Report. (XLSX)

S6 Table Interaction Network from IPA. (XLSX)
